# Enhancing Anesthetic Depth Assessment via Unsupervised Machine Learning in Processed Electroencephalography Analysis: Novel Methodological Study

**DOI:** 10.2196/77830

**Published:** 2026-02-06

**Authors:** Po-Yu Huang, Wei-Lun Hong, Hui-Zen Hee, Wen-Kuei Chang, Ching-Hung Lee, Chien-Kun Ting

**Affiliations:** 1 Department of Anesthesiology, Taipei Veterans General Hospital, No. 201, Sec 2, Shipai Rd, Beitou District, Taipei City, 11217, Taiwan, 886 228757549; 2 Institute of Electrical and Control Engineering, National Yang Ming Chiao Tung University, Hsinchu City, Taiwan; 3 Department of Anesthesiology, Far Eastern Memorial Hospital, New Taipei City, Taiwan; 4 Department of Electrical Engineering, Chung Yuan Christian University, Taoyuan City, Taiwan; 5 Institute of Emergency and Critical Care Medicine, National Yang Ming Chiao Tung University, Taipei City, Taiwan

**Keywords:** artificial intelligence, cluster analysis, electroencephalography, general anesthesia, intraoperative monitoring, machine learning

## Abstract

**Background:**

General anesthesia comprises 3 essential components—hypnosis, analgesia, and immobility. Among these, maintaining an appropriate hypnotic state, or anesthetic depth, is crucial for patient safety. Excessively deep anesthesia may lead to hemodynamic instability and postoperative cognitive dysfunction, whereas inadequate anesthesia increases the risk of intraoperative awareness. Electroencephalography (EEG)-based monitoring has therefore become a cornerstone for evaluating anesthetic depth. However, processed electroencephalography (pEEG) indices remain vulnerable to various sources of interference, including electromyographic activity, interindividual variability, and anesthetic drug effects, which can yield inaccurate numerical outputs.

**Objective:**

With recent advances in machine learning, particularly unsupervised learning, data-driven methods that classify signals according to inherent patterns offer new possibilities for anesthetic depth analysis. This study aimed to establish a methodology for automatically identifying anesthesia depth using an unsupervised, machine learning–based clustering approach applied to pEEG data.

**Methods:**

Standard frontal EEG data from participants undergoing elective lumbar spine surgery were retrospectively analyzed, yielding more than 16,000 data points. The signals were filtered with a fourth-order Butterworth bandpass filter and transformed using the fast Fourier transform to estimate power spectral density. Normalized band power ratios for delta, high-theta, alpha, and beta frequencies were extracted as input features. Fuzzy C-Means (FCM) clustering (*c*=3, *m*=2) was applied to categorize anesthetic depth into slight, proper, and deep clusters.

**Results:**

FCM clustering successfully identified 3 physiologically interpretable clusters consistent with EEG dynamics during progressive anesthesia. As anesthesia deepened, frontal alpha oscillations became more prominent within a delta-dominant background, while beta activity decreased with loss of consciousness. The fuzzy membership values quantified transitional states and captured the continuum of anesthetic depth. Visualization confirmed strong correspondence among cluster transitions, Patient State Index trends, and spectral density patterns.

**Conclusions:**

This study demonstrates the feasibility of using unsupervised machine learning to enhance anesthetic depth assessment. By applying FCM clustering to pEEG data, this approach improves the understanding of anesthesia depth and integrates effectively with existing monitoring modalities. The proposed FCM-based method complements current EEG indices and may assist anesthesia practitioners and even nonanesthesia professionals in assessing anesthetic depth to enhance patient safety.

## Introduction

General anesthesia induces temporary loss of consciousness characterized by unconsciousness, immobility, and autonomic regulation of pain [1,2]. Maintaining an appropriate depth of anesthesia is essential for patient safety. Insufficient anesthesia may lead to intraoperative awareness, autonomic hyperactivity, and significant psychological distress [3]. Conversely, excessive anesthesia has been associated with neuroinflammation [4], neuronal injury, and postoperative cognitive dysfunction or delirium [5,6]. Therefore, continuous monitoring of anesthetic depth during surgery is of critical importance. Electroencephalography (EEG)-based monitoring serves as a valuable tool for this purpose. Real-time EEG analysis provides insight into anesthetic states, as oscillatory patterns vary with the anesthetic dose, type, and patient characteristics [7,8]. The Perioperative Quality Initiative 6 Workgroup, led by Chan, details the available EEG monitors [9]. Understanding perioperative neural activity enables anesthesiologists to optimize anesthesia delivery and enhance patient outcomes.

Artificial intelligence is revolutionizing anesthesiology, with techniques such as anesthesia depth monitoring [10]. Unsupervised machine learning operates without predefined hypotheses and identifies patterns and structures within datasets to classify patients, medications, and groups [11]. Unlike supervised machine learning, which correlates the input data with known outcomes, unsupervised machine learning categorizes instances based on inherent patterns. In EEG analyses, unsupervised machine learning methods such as fuzzy clustering have been used to analyze sleep stages [12], detect epileptic spikes [13], and identify significant elements in multichannel recordings [14].

Anesthesia management fundamentally involves 3 key components: hypnosis, analgesia, and muscle relaxation. During induction and maintenance, a reproducible spectral shift occurs in the EEG, wherein frontal alpha oscillations become prominent within a delta-dominant background, delta power progressively increases with deepening anesthesia, and beta activity decreases as consciousness is lost [8,15-17]. Power spectral metrics—including relative alpha, beta, and delta power, power ratios, and spectral entropy—have been shown to reliably distinguish between conscious and anesthetized states [18-20]. Inhalational agents such as sevoflurane and desflurane consistently produce dose-dependent increases in frontal alpha and delta power [17,21-23], providing a physiologic basis for categorizing anesthesia into light, proper, and deep stages. However, current perioperative EEG-based indices, such as the Bispectral Index (BIS) [24,25] and Patient State Index (PSi) [26,27], primarily reflect the hypnotic component but can be influenced by various factors, potentially leading to misleading processed numerical outputs. Therefore, this study aimed to enhance anesthetic depth assessment by focusing on the hypnosis dimension and applying unsupervised machine learning methods, specifically Fuzzy C-Means (FCM) clustering, to processed electroencephalography (pEEG) data.

The primary objective of this study was to establish a methodology for automatically identifying anesthesia depth through a visualized presentation, leveraging machine learning–based clustering techniques. By applying unsupervised learning to pEEG data, this approach aims to enhance the classification and understanding of anesthesia depth and facilitate patient-specific anesthesia management.

## Methods

### Recruitment

Data were collected at the Veterans General Hospital, Taipei, with the approval of the Institutional Review Board (number 2021-09-008BC). More than 16,000 data points were collected from 10 participants (Participants 1‐10) who underwent elective lumbar spinal surgery in the prone position. The ages of the participants ranged from 44 to 78 years, with the American Society of Anesthesiologists’ physical status classifications ranging from class I to class III. Participants with conditions such as pregnancy, neurological disorders, ongoing infections, a history of central nervous system medication use, respiratory failure, heart failure, or renal failure were excluded.

Masimo SedLine [28] brain function monitoring was performed with the participant in the supine position. Induction included fentanyl at 1‐3 mcg/kg as an adjunct for intubation, followed by propofol at 1‐3 mg/kg as an induction bolus. Rocuronium was administered at an intubation dose of 0.6‐1 mg/kg for muscle relaxation. The participant was then intubated.

During maintenance, the inhalational agent sevoflurane was administered, with dosage adjustments titrated based on the PSi levels and pEEG density spectral array (DSA) patterns. The participant was then placed in a prone position for lumbar spinal surgery. After the completion of the surgery, the participant was returned to the supine position, and the inhalation agent was gradually titrated off. Rocuronium administration was reversed using sugammadex. Subsequently, the participants were extubated and transferred to the postanesthetic care unit for further monitoring and recovery.

### Data Collection and Processing

**Figure 1.**
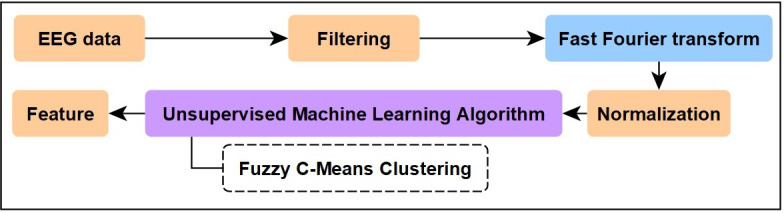
Signal processing flow diagram. Flow diagram illustrating preprocessing and feature-extraction steps applied to processed electroencephalography data obtained from participants who underwent elective lumbar spine surgery at Taipei Veterans General Hospital. Raw frontal electroencephalography signals were filtered with a fourth-order Butterworth bandpass filter, and power spectral density was estimated using the Welch method with a 30-second window and 2-second overlap. Normalized delta, high-theta, alpha, and beta band power ratios were extracted as input features for unsupervised clustering using the Fuzzy C-Means (FCM) algorithm (*c=3, m=2*). EEG: electroencephalography.

Figure 1 outlines the preprocessing steps for the EEG data in our study.

### Signal Processing and Feature Extraction

EEG tracings from the 4 Masimo SedLine channels were averaged into 1-dimensional signals and synchronized for consistency. Each participant’s EEG data were recorded at a sampling frequency of 178 Hz, with a data collection period ranging from 3 to 5 hours, depending on the duration of anesthesia. A fourth-order Butterworth bandpass filter (0.5‐30 Hz) was applied to minimize the ripple and achieve a maximally flat response. Power spectral density was estimated using the Welch method implemented in Python (SciPy library), using a 30-second window with a 2-second sliding overlap to ensure both temporal continuity and frequency resolution. Additional preprocessing specifications are available in Multimedia Appendix 1. The Welch method and the fast Fourier transform were used to estimate the power spectral density, facilitating the extraction of alpha (8‐12 Hz), beta (13‐30 Hz), delta (0.5‐4 Hz), and high-theta (6‐8 Hz) band powers. Normalization was performed by calculating the ratio of each band’s power to the sum of the power of these 4 specific bands (delta+high-theta+alpha+beta), ensuring that the sum of the feature vector elements equals 1. The 4‐6 Hz and 12‐13 Hz bands were excluded based on their limited variation across states. Removing these transitional zones established a “spectral buffer” to reduce noise, allowing the algorithm to prioritize the more discriminative delta and alpha and high-theta features. Manual artifact rejection was conducted by board-certified anesthesiologists and engineers to identify and exclude segments exhibiting abnormal, noisy epochs indicative of artifacts.

### FCM Clustering Algorithm

FCM clustering categorizes data points into clusters with membership values between 0 and 1, accommodating the uncertainty in the cluster assignment. It was performed with 3 clusters (*c*=3), a fuzziness parameter (*m*) of 2, random initialization of cluster centers, and a convergence threshold of 0.005. The model iteratively updates the centroids and membership values until convergence, guided by a fuzziness parameter (*m*) that regulates cluster ambiguity. A global FCM model was established to generate fixed global cluster centroids. These fixed centroids were then applied to all participants to calculate membership values. Further implementation details are provided in Multimedia Appendix 1. This method extracts delta, alpha, high-theta, and beta band power ratios from pEEG signals to partition the data into 3 statistically distinct clusters. These clusters were subsequently labeled as “slight,” “proper,” and “deep” based on their physiological spectral characteristics. The capability of FCM to manage data ambiguity makes it suitable for nuanced depth assessments under general anesthesia.

### Ethical Considerations

This study was conducted in accordance with the ethical principles outlined in the Declaration of Helsinki and was approved by the Institutional Review Board of Taipei Veterans General Hospital, Taiwan (number: 2021-09-008BC). Written informed consent was obtained from all participants prior to enrollment. The data used in this study were deidentified before analysis to ensure participant confidentiality and privacy. No identifiable personal information or images were included in the manuscript or supplementary materials. Participants did not receive any financial compensation for their participation in the study.

## Results

### Overview

In this study, an unsupervised learning method, FCM, was used to analyze the pEEG data from participants with complete datasets. Table 1 summarizes the demographic information and baseline characteristics of the participants. The analysis focused on a feature set comprising the delta-band power ratio and the sum of the alpha and high-theta band power ratios. The results revealed 3 distinct clusters. Based on the centroids’ spectral features, we assigned post hoc clinical labels to these clusters representing different anesthesia depths: slight (green), proper (blue), and deep (red). This clustering approach was validated using visual representation to confirm the effectiveness of the feature set in differentiating anesthesia depths. Subsequently, the same method was applied to individual participant data, categorizing each participant’s pEEG into 1 of the 3 clusters, thus providing a detailed estimation of the anesthesia depth for each individual.

**Table 1.** Demographic information and baseline characteristics. Demographic and intraoperative characteristics of 10 participants included in this retrospective observational study of processed electroencephalography–based anesthetic-depth assessment. All participants underwent elective lumbar spine surgery at Taipei Veterans General Hospital, Taiwan. Variables include age, sex, height, weight, BMI, American Society of Anesthesiologists classification, comorbidities, intraoperative blood loss, operation time, and postoperative hospital stay.

Participant	Age (years)	Sex	Height (cm)	Body weight (kg)	BMI (kg/m^2^)	ASA^a^ classification	Comorbidities	Blood loss (mL)	Operation time (minutes)	Postoperative stay (days)
1	63	F^b^	152.2	63.7	27.5	I	None	300	250	7
2	78	F	150.5	53.5	23.6	II	Hepatitis	200	280	6
3	65	M^c^	168	89.8	31.8	II	None	300	360	4
4	75	M	170	70	24.2	II	Hypertension, dementia	660	405	7
5	67	M	160	64	25.0	II	Hypertension	200	285	6
6	45	M	171	69.9	23.9	I	None	^d^—	170	2
7	61	M	160.3	77.7	30.2	II	Hypertension, diabetes mellitus	1300	195	6
8	68	F	157.7	71.1	28.6	II	Hypertension, diabetes mellitus	450	210	5
9	44	M	173.6	67.6	22.4	I	None	50	375	29
10	71	M	167	83	29.8	III	Hypertension, coronary artery disease	530	320	6

a ASA: American Society of Anesthesiologists.

b F: female.

c M: male.

d Not available.

### FCM Clustering Analysis of pEEG Features

**Figure 2.**
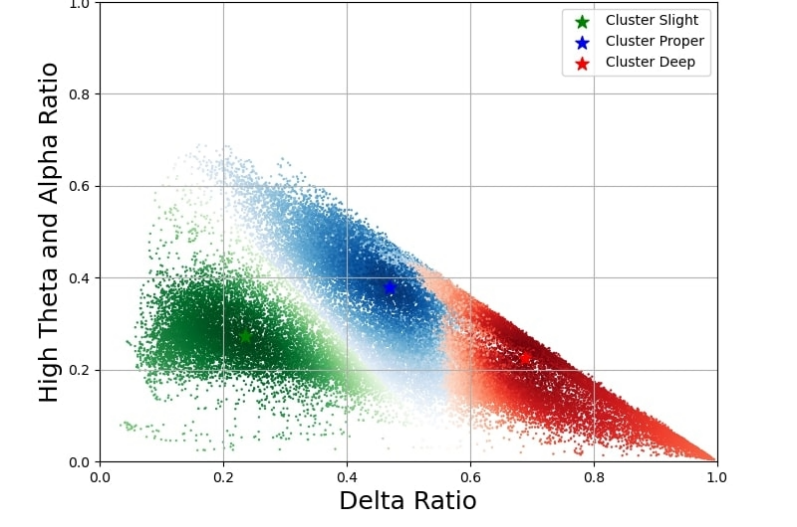
Fuzzy C-Means clustering results for all participants. Two-dimensional clustering distribution of processed electroencephalography features from participants undergoing elective lumbar spine surgery. The *x*-axis represents the delta-power ratio and the *y*-axis the combined alpha and high-theta power ratios. Fuzzy C-Means clustering (*c=3, m=2*) identified 3 physiologically interpretable clusters corresponding to anesthesia-depth levels: slight (green), proper (blue), and deep (red). Each point denotes a 30-second windowed segment of electroencephalography data, demonstrating the continuum of anesthetic states observed across the study population.

FCM clustering was applied to all participants, using alpha, beta, delta, and high-theta band power as the selected features for clustering. Figure 2 illustrates the FCM clustering results for all participants, with the delta band power ratio plotted on the *x*-axis and the sum of the alpha and high-theta band power ratios on the *y*-axis. Each data point represents a single time point of a participant’s pEEG signal and is clustered into 3 distinct groups: slight anesthesia depth (green), proper anesthesia depth (blue), and deep anesthesia depth (red). The contribution of the beta band power ratio can be inferred as the sum of the delta, alpha, high-theta, and beta band power ratios equals 1, although not explicitly displayed. This visual depiction offers insights into the distribution of a participant’s single time points within each anesthesia depth cluster.

After establishing the efficacy of the selected features and parameters through a collective analysis of the participants, our investigation shifted focus to the individual application of this methodology. The fixed global cluster centroids were then applied to the data of Participants 1‐10 to calculate membership values and simplified estimation results. Following the methodology outlined, where all time indices share a common time scale, the pEEG data were combined with PSi data retrieved from the Masimo SedLine brain function monitoring system, and the time-frequency images were compared within the same figure, with each participant outputting a distinct figure. Within these figures, Participants 4 and 6 are described in the article. By incorporating this comprehensive approach, our study verified the applicability of the selected features and enhanced confidence in the adaptability of our methodology to diverse clinical scenarios and datasets, strengthening the reliability of our conclusions and paving the way for further exploration and validation within the anesthesia depth assessment domain.

### Participant 4 and Participant 6: FCM Clustering Analysis

**Figure 3.**
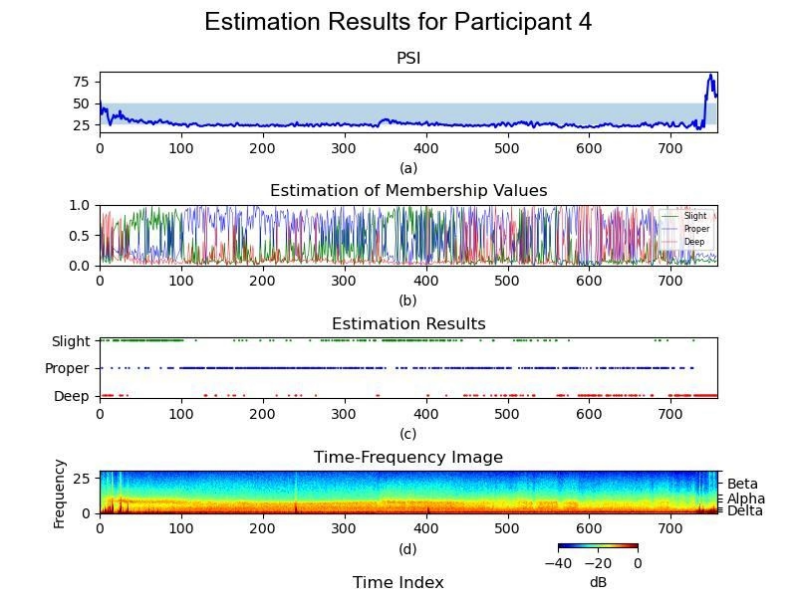
Fuzzy C-Means clustering analysis of Participant 4. Representative analysis of Participant 4 (male, 75 years, American Society of Anesthesiologists class II) who underwent lumbar spine surgery at Taipei Veterans General Hospital. (**A**) shows the Patient State Index trend over time; (**B**) displays Fuzzy C-Means membership values representing the degree of belongingness to each cluster; (**C**) illustrates the simplified classification into slight, proper, and deep anesthesia levels; (**D**) presents the corresponding electroencephalography time-frequency spectrogram. These plots depict temporal transitions in anesthetic depth.

Participant 4 underwent the same data retrieval and processing procedures as the other participants, with raw EEG data obtained from the Masimo SedLine brain function monitoring system. These data were subjected to filtering, fast Fourier transformation, normalization, and subsequent FCM clustering. The culmination of these processes is depicted in Figure 3, which comprises multiple panels, sharing a synchronized timescale on the *x*-axis.

Figure 3A shows the PSi of Participant 4 obtained from the Masimo SedLine brain function monitoring system. The *x*-axis represents the time scale, whereas the *y*-axis displays the PSi values, reflecting the PSi over time.

Figure 3B shows the results of FCM clustering for Participant 4. The *y*-axis illustrates the estimated membership values and denotes the degree of belongingness of each cluster. This belongingness quantifies the “fuzziness” of the FCM clustering, indicating the confidence level of each assignment of the data point to a particular cluster. It effectively quantifies the “color shading” shown in Figure 2, where darker shades represent higher confidence levels in cluster assignments. For example, a membership value of 1 signifies full belongingness to the cluster, and a value of 0.8 indicates 80% confidence in the assignment. This “soft” labeling approach allows for a more nuanced understanding of the clustering results, providing insights into the uncertainty associated with each classification of the data point.

Figure 3C represents a modified rendition of Figure 3B, specifically tailored to illustrate the anesthesia depth classification for Participant 4. Along the *y*-axis, this plot delineates the categorization into slight, proper, or deep anesthesia groups at individual time points. Figure 3B provides a detailed depiction of the continuous membership values, reflecting the degree of belonging to each cluster: slight, proper, and deep. However, this comprehensive display introduces the challenge of intuitively discerning the predominant cluster at a single point. While this approach offers a more complete dataset, it complicates the task of determining the cluster affiliations for each data point. Hence, the modified version in Figure 3C simplifies this interpretation by highlighting the cluster with the “higher value.” This adaptation facilitates a more straightforward assessment of the anesthesia depth classification by presenting a clearer distinction. This clarity enhances the utility of visualization in aiding clinician decision-making during patient monitoring and management.

Figure 3D presents a time-frequency image derived from the raw EEG signal without processing, providing insights into the frequency components of the EEG signal over time. Overall, Figure 3 provides a comprehensive analysis of the EEG signals and anesthesia depth classification for Participant 4, contributing to the understanding of brain function monitoring and anesthesia management.

**Figure 4.**
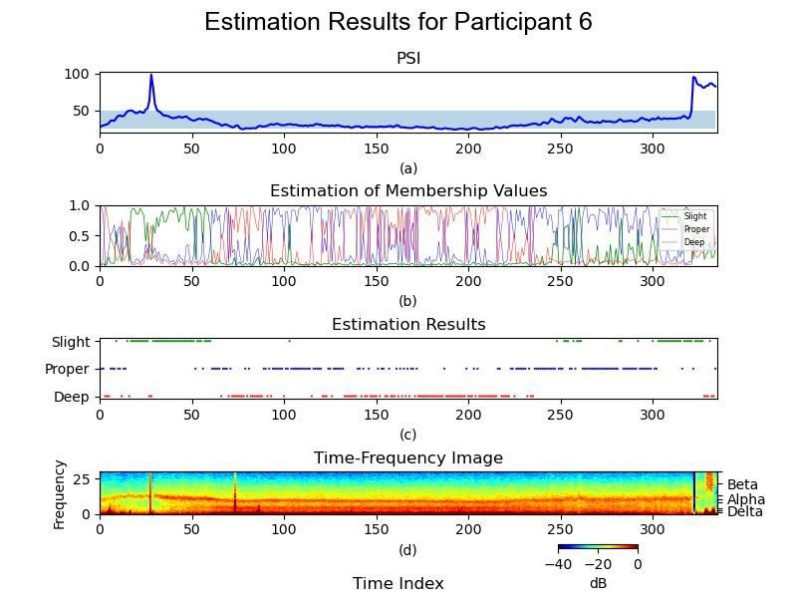
Fuzzy C-Means clustering analysis of Participant 6. Representative analysis of Participant 6 (male, 45 years, American Society of Anesthesiologists class I) who underwent elective lumbar spine surgery at Taipei Veterans General Hospital. (**A**) shows the Patient State Index trend over time. (**B**) displays Fuzzy C-Means membership values representing the degree of belongingness to each cluster. (**C**) illustrates the simplified classification into slight, proper, and deep anesthesia levels. (**D**) presents the corresponding electroencephalography time-frequency spectrogram. These plots depict temporal transitions in anesthetic depth. PSi: Patient State Index.

Participant 6 then underwent identical signal processing and analysis procedures, resulting in Figure 4. This comprehensive Figures 4A-D underwent the same rigorous process as shown in Figures 3A-D.

## Discussion

### Principal Findings

This study explored the feasibility of improving anesthetic depth assessment by applying unsupervised machine learning to pEEG data. EEG patterns vary with the depth of anesthesia [10]. PEEG-derived indices, such as the BIS and PSi, are susceptible to various influences, presenting limitations in their interpretation [24]. Purdon et al [8] characterized the EEG signatures of intravenous and inhaled anesthetics, providing additional insights into the depth of anesthesia. In Figure 2, the delta ratio quantifies cortical suppression, as delta oscillations reflect deep anesthesia and thalamocortical inhibition. The theta and alpha ratios were selected for their modulation by anesthetics in the frontal cortex. Alpha oscillations indicate stable unconsciousness under propofol and sevoflurane, correlating with GABAergic inhibition, while theta oscillations become prominent at higher anesthetic concentrations, particularly with sevoflurane, signaling transitions between moderate and deep anesthesia. Figures 3 and 4 depict Participants 4 and 6, respectively, demonstrating the combination of PSi values, anesthesia depth clustering values, simplified clustering results, and processed time-frequency images. These visual presentations illustrate patient-specific anesthesia depth, facilitating the automatic identification of anesthesia depth.

Postoperative delirium and neurocognitive complications after spinal surgery remain significant concerns [29], and perioperative EEG monitoring may contribute to their management [30]. In this study, one participant (Participant 4) had a history of cognitive dysfunction due to dementia. All participants underwent lumbar spinal surgery in the prone position with sevoflurane as the maintenance agent. The operation time ranged from 170 to 405 minutes. Most participants were discharged within 1 week, and no other cognitive impairments were documented.

### Limitations

This study was constrained by a small sample size. While the extensive dataset of over 16,000 EEG epochs ensures high within-subject data density and supports the internal stability of the model for the analyzed participants, we acknowledge that the limited number of independent participants restricts the external generalizability of the findings. Previous studies have indicated that a sampling frequency of 100 Hz is sufficient for EEG-based analysis [31]. While a larger sample size could further refine the FCM clustering results presented in Figure 1, the primary determinants of clustering effectiveness in single-subject EEG analysis are the duration and density of the data. With 3 to 5 hours of continuous EEG recordings per participant, our study provided sufficient granularity for meaningful intrasubject clustering, as demonstrated in Figures 3 and 4. Our primary objective was to explore the feasibility and establish the methodological framework for applying unsupervised machine learning to processed EEG data.

García et al [32] explored EEG activity and delineated an analgesia-related axis alongside hypnosis during general anesthesia, revealing distinct changes in EEG patterns. We acknowledge the limitation of a single-dimensional index in capturing the full spectrum of neural activity during anesthesia. However, our primary goal is to demonstrate the feasibility of unsupervised clustering within a clinical framework, focusing on the hypnosis axis—one of anesthesia’s 3 key components. This study used frontal EEG signals, as used in processed systems such as BIS and SedLine. Frontal EEG monitoring inherently limits spatial coverage but remains the clinical standard due to its accessibility and reliability. The frontal cortex exhibits predictable EEG dynamics—α-spindle emergence and δ-wave dominance—with increasing anesthetic depth, correlating with loss of consciousness and hypnotic adequacy [33-35].

While this study does not include external validation or direct quantitative comparisons, this reflects the broader challenge in anesthetic depth classification, which lacks a universal standard. Although no intraoperative recall was observed, determining the optimal depth of anesthesia remains complex. Conventional monitors, such as BIS and SedLine, along with DSA visualization, aid clinical judgment but do not serve as definitive references. The study introduces a complementary tool to enhance anesthetic depth assessment. This is reflected in Figures 3 and 4, where multiple monitoring modalities are integrated to provide additional insights.

Finally, the frame-by-frame analysis maximizes temporal sensitivity but may result in rapid state transitions, or “flickering,” as observed in Figure 3C. For clinical application, future iterations would likely require temporal smoothing algorithms to stabilize the output and minimize alarm fatigue. Despite strong visual correspondence with PSi trends, standardized quantitative metrics were not calculated due to methodological constraints. Specifically, the significant time delay inherent in the PSi algorithm [36] limits direct linear correlation with the frame-by-frame FCM method, and imposing rigid thresholds for concordance rates contradicts the continuous, fuzzy nature of anesthetic depth.

### Comparison With Prior Work

Unsupervised learning involves algorithms that identify patterns or structures within a dataset, facilitating the discovery of novel classification methods [10]. Systematic reviews have highlighted the effectiveness of unsupervised clustering methods in various medical applications. Clustering analysis has revealed pivotal features underlying the progression from early stage to advanced Alzheimer disease [37]. Unsupervised learning models applied in cardiac resynchronization therapy can accurately discern patient clusters and are valuable for phenotyping and predicting treatment responses [38]. In amyotrophic lateral sclerosis research, the k-means score, a hierarchical clustering method, has emerged as the most widely used method for stratification and progression prediction [39]. We predefined 3 practical categories—slight, proper, and deep anesthesia—to emulate the structured reasoning process that governs intraoperative anesthetic management. Clinicians continuously evaluate anesthetic depth and determine whether to increase, maintain, or decrease anesthetic delivery. Each titration is typically incremental and followed by a brief observation period to allow pharmacokinetic redistribution and physiological stabilization before reevaluation. Although the equilibration interval varies among individuals, such judgments integrate both the instantaneous anesthetic state and its temporal evolution [36]. FCM clustering offers distinct advantages for modeling this continuum. It enables classification into clinically interpretable states while concurrently generating fuzzy membership values that quantify the degree to which a given epoch inclines toward a “lighter” or “deeper” level of anesthesia. This property captures the continuous and overlapping nature of anesthetic depth and mirrors the gradual, feedback-driven adjustments that characterize intraoperative anesthesia management.

In our continuous monitoring data, transitions between anesthetic states occurred gradually, consistent with the physiological continuum of anesthesia depth. Abrupt shifts from “light” directly to “deep” anesthesia were uncommon and, when observed, were generally attributable to identifiable physiological perturbations—such as bolus drug administration, intense noxious stimulation, or sudden hemodynamic changes—rather than modeling artifacts. Processed EEG indices may also be transiently affected by electromyographic [40], electrocardiographic [41], or mechanical vibration artifacts, particularly during spine surgery. These interferences are usually recognizable in the raw EEG tracing and can be excluded from analysis.

Near-equal memberships across clusters (≈33% each) are uncommon and typically occur when a feature vector lies between centroids or during transient noise, reflecting a transitional or indeterminate phase rather than a distinct anesthetic state. Interpretation of anesthetic depth is inherently pattern-based, as clinicians assess temporal trends and spectral features in the DSA—for instance, alpha dominance suggests adequate hypnosis, whereas delta and theta predominance or burst suppression indicate a deeper anesthetic level [42]. Therefore, a single epoch with near-equal memberships would not influence clinical judgment, which relies on the overall evolution of EEG patterns rather than isolated points.

Prior research has focused on developing and enhancing automated system controls, including closed-loop anesthesia. Components such as measurement devices, controllers, and actuators are central to these advancements [43]. pEEG-derived indices, such as the BIS [43,44] and PSi [45], frequently serve as key targets. This study introduced unsupervised learning clustering as a potential new approach for assessing anesthesia depth, offering additional avenues for improving automated anesthesia control systems.

The novel clustering approach provides an alternative method for assessing anesthesia depth, particularly focusing on the “hypnosis” axis, as depicted in Figure 3B and C. These figures illustrate the EEG density spectral array data from Participant 4, suggesting segmentation into slight, proper, and excessively deep anesthesia depths. Unlike traditional PSi values, which provide singular metrics, the representation of Figure 3B with fuzziness on the *y*-axis shows nuanced membership toward anesthesia depth clusters. The graphical depiction in Figure 3C highlights the peak values, indicating the period of deep anesthesia. This approach offers a novel tool for assisting clinical judgment in anesthesia depth assessment.

Among conventional unsupervised clustering paradigms, FCM, Gaussian Mixture Models (GMMs), and density-based algorithms such as Density-Based Spatial Clustering of Applications with Noise (DBSCAN) exhibit fundamentally distinct analytical mechanisms. FCM minimizes a distance-weighted objective function while permitting graded memberships across clusters—an essential property for anesthesia-related data in which EEG and physiological variables vary continuously and overlap across anesthetic states. In contrast, GMM represents data as a superposition of Gaussian components and assumes that each cluster conforms to an elliptical, symmetric distribution. However, anesthetic EEG features often display non-Gaussian characteristics, including skewness and heavy tails arising from nonlinear pharmacodynamic effects and interindividual variability, rendering GMM less robust and prone to boundary instability. Density-based algorithms such as DBSCAN, although effective in identifying discrete or spatially separated clusters, depend on fixed density thresholds and therefore perform poorly with continuous-depth data, where transitions between anesthetic states are gradual and density gradients evolve smoothly. Considering these properties, FCM provides a mathematically rigorous and physiologically consistent framework for delineating the smooth, overlapping transitions that define anesthetic depth.

Building upon these theoretical considerations, we initially explored k-means clustering but found it less flexible in classifying anesthetic depth. When visualizing frequency band proportions, k-means struggled to capture transition zones effectively, reinforcing our decision to use FCM for a more adaptable classification. The FCM algorithm stands out in unsupervised machine learning because of its adaptability and effectiveness in increasing data volumes. It iteratively adjusts centroids and clusters, creating personalized representations tailored to individuals, analogous to bespoke garments [46]. This adaptability ensures an accurate assessment of anesthesia depth dynamics, which is crucial for personalized healthcare.

### Conclusions

In conclusion, this study established a machine learning–based methodology for anesthesia depth assessment, demonstrating its feasibility and providing preliminary insights into classification, visualization, and patient-specific management. By applying FCM clustering to processed EEG data, this novel approach enhances the understanding of anesthesia depth patterns and integrates with existing monitoring modalities, including PSi values and time-frequency image visualization for a more comprehensive assessment. Although preliminary, these findings lay the foundation for future research and refinement.

## Supplementary material

10.2196/77830 Multimedia Appendix 1 Detailed data preprocessing workflow and Fuzzy C-Means (FCM) parameter settings. Electromyography signals were processed via the Welch method (*30-s windows, 2-s overlap*). Normalized delta, high-theta, alpha, and beta band-power ratios were extracted as input features. The FCM algorithm (*c = 3, m = 2*) was applied with random initialization of cluster centers to identify three anesthesia depth clusters. Measures of dispersion for the delta band within each cluster are summarized in Table S1. Figures S1 and S2 show additional two-dimensional clustering distributions, demonstrating consistent three-cluster separation across different frequency-band pairings.

## Abbreviations

BIS: Bispectral Index
DBSCAN: Density-Based Spatial Clustering of Applications with Noise
DSA: density spectral array
EEG: electroencephalography
EMG: electromyography
FCM: Fuzzy C-Means
GMM: Gaussian Mixture Model
pEEG: processed electroencephalography
PSi: Patient State Index
