# Supplementary material for: Enhancing Anesthetic Depth Assessment via Unsupervised Machine Learning in Processed Electroencephalography Analysis: Novel Methodological Study
Source: JMIR Med Inform. 2026 Feb 6;14:e77830. doi: 10.2196/77830 (PMC12880611; doi:10.2196/77830)

Multimedia Appendix 1: Detailed data preprocessing and Fuzzy C-Means parameter settings

This supplementary file comprises two parts:

1. **Data preprocessing**
2. **Fuzzy C-Means parameter configuration**.

Part 1. Data Preprocessing

Welch's method was implemented using the *welch* function from the *scipy.signal* module in Python (*SciPy package*), and the Fuzzy C-Means clustering algorithm was implemented using the *cmeans* function within the *skfuzzy.cluster* submodule of the *scikit-fuzzy* Python package.

For Welch's method, the segment length (*nperseg*) was determined by multiplying the raw EEG sampling frequency by 30 seconds, corresponding to the time window used for pEEG index generation. A 2-second sliding window was applied for overlap. Regarding frequency resolution, analysis of the delta band requires approximately 1 Hz resolution to accurately visualize power distribution.

Part 2. Fuzzy C-Means Clustering Parameters

Three clusters ($c = 3$) were used, with the fuzziness parameter (m) set to the commonly adopted value of 2. The maximum number of iterations was set to 100; however, in ten separate runs, the algorithm typically converged—fulfilling the objective function—after approximately 30 iterations. The initial cluster centers were randomly initialized by the *skfuzzy* function. The stopping criterion was set to 0.005, meaning that the algorithm terminated when the norm of the change in the fuzzy partition matrix fell below this threshold.

The number of data points in each cluster was as follows: (*Slight, 10,737; proper, 24,462; and deep, 24,671*).

The cluster center values (power ratios of δ, θ, α, and β bands) were:

1. Slight: (0.235, 0.067, 0.206, 0.491)
2. Proper: (0.469, 0.108, 0.271, 0.151)
3. Deep: (0.688, 0.111, 0.115, 0.0856)

Measures of dispersion for the δ band within each cluster are summarized in *Supplementary Table S1*.

Supplementary Table S1

Band	Cluster	Mean	Median	Std Dev	Min	Max	Skewness	Kurtosis
**δ**	Slight	0.242	0.227	0.098	0.034	0.549	0.4047	-0.777
	Proper	0.461	0.474	0.081	0.128	0.614	-0.751	0.204
	Deep	0.688	0.664	0.102	0.498	0.994	1.0369	0.470
**θ + α**	Slight	0.265	0.260	0.074	0.024	0.646	0.473	1.551
	Proper	0.376	0.390	0.100	0.072	0.689	-0.270	-0.120
	Deep	0.223	0.233	0.095	0.004	0.431	-0.311	-0.801
**β**	Slight	0.492	0.488	0.093	0.231	0.887	0.377	0.147
	Proper	0.162	0.146	0.078	0.039	0.393	0.644	-0.572
	Deep	0.088	0.062	0.063	0.001	0.407	1.353	1.039

To further validate clustering consistency, additional two-dimensional clustering plots were generated, comparing the beta power ratio (X-axis) with the combined high-theta and alpha ratio (Y-axis), and the delta power ratio (X-axis) with the combined high-theta and alpha ratio (Y-axis). As shown in *Supplementary Figures S1 and S2*, the resulting distributions exhibited a comparable three-cluster separation pattern, reinforcing the stability of the FCM model across different frequency-band pairings.

Supplementary Figure S1

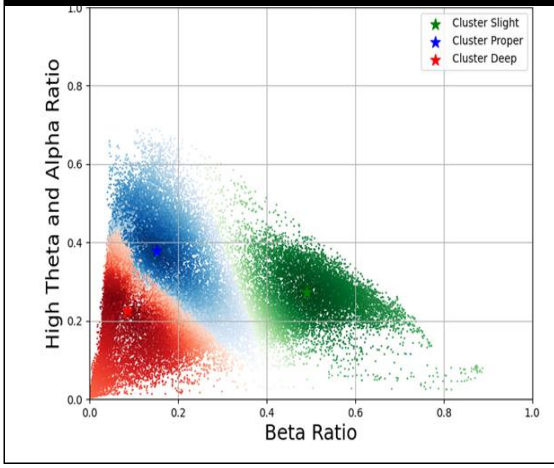

Supplementary Figure S2

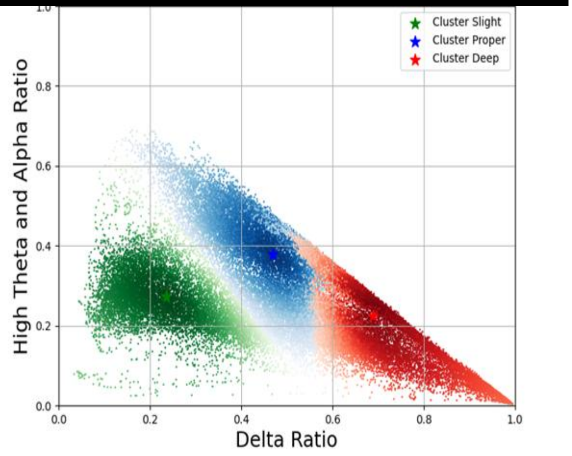

Supplement: Multimedia Appendix 1 [file medinform-v14-e77830-s001.pdf]
